# G-protein-coupled receptor 40 agonist GW9508 potentiates glucose-stimulated insulin secretion through activation of protein kinase C*α* and *ε* in INS-1 cells

**DOI:** 10.1371/journal.pone.0222179

**Published:** 2019-09-09

**Authors:** Takuya Hashimoto, Hideo Mogami, Daisuke Tsuriya, Hiroshi Morita, Shigekazu Sasaki, Tatsuro Kumada, Yuko Suzuki, Tetsumei Urano, Yutaka Oki, Takafumi Suda

**Affiliations:** 1 2nd Department of Internal Medicine, Hamamatsu University School of Medicine, Shizuoka, Japan; 2 Department of Health and Nutrition, Tokoha University, Shizuoka, Japan; 3 Department of Occupational Therapy, Tokoha University, Shizuoka, Japan; 4 Department of Medical Physiology, Hamamatsu University School of Medicine, Shizuoka, Japan; 5 Department of Family and Community Medicine, Hamamatsu University School of Medicine, Shizuoka, Japan; Indiana University School of Medicine, UNITED STATES

## Abstract

**Objective:**

The mechanism by which G-protein-coupled receptor 40 (GPR40) signaling amplifies glucose-stimulated insulin secretion through activation of protein kinase C (PKC) is unknown. We examined whether a GPR40 agonist, GW9508, could stimulate conventional and novel isoforms of PKC at two glucose concentrations (3 mM and 20 mM) in INS-1D cells.

**Methods:**

Using epifluorescence microscopy, we monitored relative changes in the cytosolic fluorescence intensity of Fura2 as a marker of change in intracellular Ca^2+^ ([Ca^2+^]_*i*_) and relative increases in green fluorescent protein (GFP)-tagged myristoylated alanine-rich C kinase substrate (MARCKS-GFP) as a marker of PKC activation in response to GW9508 at 3 mM and 20 mM glucose. To assess the activation of the two PKC isoforms, relative increases in membrane fluorescence intensity of PKC*α*-GFP and PKC*ε*-GFP were measured by total internal reflection fluorescence microscopy. Specific inhibitors of each PKC isotype were constructed and synthesized as peptide fusions with the third α-helix of the homeodomain of Antennapedia.

**Results:**

At 3 mM glucose, GW9508 induced sustained MARCKS-GFP translocation to the cytosol, irrespective of changes in [Ca^2+^]_*i*_. At 20 mM glucose, GW9508 induced sustained MARCKS-GFP translocation but also transient translocation that followed sharp increases in [Ca^2+^]_*i*_. Although PKC*α* translocation was rarely observed, PKC*ε* translocation to the plasma membrane was sustained by GW9508 at 3 mM glucose. At 20 mM glucose, GW9508 induced transient translocation of PKC*α* and sustained translocation as well as transient translocation of PKC*ε*. While the inhibitors (75 μM) of each PKC isotype reduced GW9508-potentiated, glucose-stimulated insulin secretion in INS-1D cells, the PKC*ε* inhibitor had a more potent effect.

**Conclusion:**

GW9508 activated PKC*ε* but not PKC*α* at a substimulatory concentration of glucose. Both PKC isotypes were activated at a stimulatory concentration of glucose and contributed to glucose-stimulated insulin secretion in insulin-producing cells.

## Introduction

Diabetes is reaching pandemic proportions and is expected to affect over 592 million people worldwide by 2035 [1]. The most common form of this disease, type 2 diabetes, develops when pancreatic *β*-cells are unable to secrete an adequate amount of insulin to compensate for peripheral insulin resistance [2]. Insulin secretion is regulated chiefly by glucose through intracellular metabolism and is also modulated by non-glucose secretagogues, such as incretin hormones and fatty acids, and muscarinic stimulation via G protein-coupled receptors (GPCRs) [3]. Because activation of these receptors augments insulin secretion only when glucose levels are elevated, they can be targeted therapeutically to raise insulin levels with minimal risk of iatrogenic hypoglycemia.

Glucagon-like peptide-1 (GLP-1) potentiates glucose-stimulated insulin secretion (GSIS) by binding to its GPCRs in *β*-cells [4,5]. GLP-1 receptor agonists are clinically available and pose little risk of iatrogenic hypoglycemia. GLP-1 potentiates GSIS by increasing cyclic adenosine monophosphate (cAMP) levels, leading to activation of protein kinase A (PKA) [6]. Conversely, in our previous study and a recent report from another group, GLP-1 also stimulated insulin secretion by activating protein kinase C (PKC) [7,8].

Ten isoforms of PKC have been identified and divided into three major classes, based on their requirements for activators. Conventional PKC (cPKC; PKC*α*, PKC*β*I, PKC*β*II, and PKC*γ*) is activated by both Ca^2+^ and diacylglycerol (DAG); novel PKC (nPKC; PKC*δ*, PKC*ε*, PKC*η*, and PKC*θ*) requires only DAG; and atypical PKC (aPKC; PKC*ζ* and PKC*λ*) binds neither Ca^2+^ nor DAG [9]. DAG, which is essential for the activation of cPKC and nPKC, is mainly derived from the hydrolysis of plasma membrane phosphatidylinositol 4,5-bisphosphate (PIP_2_). This hydrolysis is due to activation of phospholipase C (PLC), following the binding of an agonist to a GPCR. Previously, we showed that GLP-1 activated an nPKC (PKC*ε*) predominantly via PLC activation, rather than a cPKC (PKC*α*) in INS-1 cells, an insulin-secreting cell line [7]. However, it was insufficient to assess how PLC/PKC signaling alone was involved in GSIS, because GLP-1 must activate not only PLC/PKC signaling but also cAMP/PKA signaling.

G-protein-coupled receptor 40 (GPR40) belongs to the free fatty acid receptor family [10–12]. Several reports have shown that GPR40 couples mainly to G*α*_q_/G*α*_11_, which activates PLC, resulting in the formation of inositol 1,4,5-trisphosphate (IP_3_) and DAG via PIP_2_ hydrolysis [13]. IP_3_ induces Ca^2+^ release from the endoplasmic reticulum (ER), and DAG activates PKC [14]. The natural ligands of GPR40 include medium- and long-chain free fatty acids [15,16]. GPR40 is preferentially expressed in pancreatic islets and *β*-cell lines [15,17], and its expression in human pancreatic islets appears to be higher than that of sulfonylurea receptor type 1 (SUR1) and GLP-1 receptor (GLP-1R) [17,18]. GPR40 contributes to approximately half of the insulin secretory response to fatty acids [19]. Activation of GPR40 significantly enhances GSIS from rodent pancreatic islets and *β*-cell lines [15,20–23]. However, reports have never been clear that PKC is involved in the increase in GSIS via GPR40 signaling, nor has GPR40-mediated PKC activation been examined at the isoform level.

We evaluated the role of the PKC pathway in the enhancement of GSIS by GPR40 activation. To analyze this, we chose GW9508, a selective and potent small-molecule agonist of GPR40 [21]. Among the multiple PKC isoforms that are expressed in pancreatic *β*-cells, PKC*α* and PKC*ε* are likely to have dominant functions in GSIS [24,25]. The roles of these two proteins in GW9508-potentiated GSIS were also determined. To minimize the interference of glucose against GW9508-induced signal transduction, we conducted this study in INS-1 cells, which secrete less insulin in response to glucose stimulation than primary *β*-cells.

## Materials and methods

### Plasmid construction

PKC*α*-pEGFP was obtained from Clontech (Palo Alto, CA, USA). The plasmids encoding PKC*ε*-GFP and MARCKS-GFP were prepared as described previously [26,27].

### Cell culture and transfection

INS-1D cells were a gift from Dr. Sekine (Tokyo University) [28]. The cells were grown in 60-mm culture dishes at 37°C and 5% CO_2_ in a humidified atmosphere. The culture medium was RPMI 1640 (Sigma, St. Louis, MO, USA) supplemented with 10 mM glucose, 10% fetal bovine serum, 1 mM sodium pyruvate, 1 mM L-glutamine, and 50 μM 2-mercaptoethanol. For fluorescence imaging, the cells were cultured in a 35-mm glass-bottom dish (AGC Techno Glass Co., Ltd., Shizuoka, Japan) at 50% confluence 2 days before transfection. A plasmid encoding the GFP-tagged proteins was transfected into the cells using Lipofectamine 2000 (Invitrogen, Burlington, ON, Canada). Experiments were performed within 2 days of transient transfection. We established stable transfectants from parental INS-1 cells expressing myristoylated alanine-rich C kinase substrate (MARCKS)-GFP or PKC*α*-GFP by G418 selection and cloning.

### Solutions

The standard extracellular solution contained 140 mM NaCl, 5 mM KCl, 1 mM MgCl_2_, 2.5 mM CaCl_2_, 3 mM glucose, and 10 mM Hepes-NaOH (pH 7.3). The solution for membrane depolarization contained 105 mM NaCl, 40 mM KCl, 1 mM MgCl_2_, 2.5 mM CaCl_2_, 3 mM glucose, and 10 mM Hepes-NaOH (pH 7.3). For the high-glucose condition, 20 mM glucose replaced 3 mM glucose in the extracellular solution. The cells, placed in a glass-bottom dish, were perfused continuously from a gravity-fed system. Experiments were performed in the standard extracellular solution at room temperature, unless otherwise noted. Krebs-Ringer buffer (KRB) contained 119 mM NaCl, 4.6 mM KCl, 1 mM MgSO_4_, 0.15 mM Na_2_HPO_4_, 0.4 mM KH_2_PO_4_, 25 mM NaHCO_3_, 2 mM CaCl_2_, and 20 mM Hepes-NaOH (pH 7.3).

### Reagents

GW9508 was purchased from Focus Biomolecules (Plymouth Meeting, PA, USA). Gö 6976 and bisindolylmaleimide I (BIS I) were from Calbiochem (La Jolla, CA, USA). Fura2 (acetoxymethyl form, AM) was obtained from Invitrogen. All other chemicals were acquired from Sigma. A PKCα inhibitory peptide, antennapedia-PKC-(19–31) (antp-PKC*α*; RRMKW KKRFA RKGAL RQKNV), and a PKC*ε* inhibitory peptide, antennapedia-PKC-(149–164) (antp-PKC*ε*; RRMKW KKERM RPRKR QGAVR RRV), were synthesized by Medical and Biological Laboratories Co., Ltd. (Nagoya, Japan). These inhibitory peptides are synthesized in tandem and comprise amino acids from the third α-helix of the homeodomain of Antennapedia (antp; residues 52–58, which regulate penetration) [29][30] and the PKCα (residues 19–31) or PKCε (residues 149–164) pseudosubstrate peptides [31][32]. Antennapedia, used as a control, was also synthesized by Medical and Biological Laboratories Co., Ltd.

### Imaging experiments

#### Epifluorescence microscopy

Fluorescence images were captured at 5-s intervals using a Nikon inverted microscope (60×/1.45 numerical aperture oil immersion objective) that was equipped with a cooled (−85°C) charge-coupled-device digital camera, and recorded and analyzed on a NIS-Elements imaging station (Nikon Corporation, Tokyo, Japan). The excitation light source was a 150-watt xenon lamp with a high-speed scanning polychromatic light source. GFP fluorescence was excited at 488 nm, and the emitted light was collected through a 535/45-nm bandpass filter with a 505-nm dichroic mirror. We measured the fluorescence intensity of the GFP-tagged proteins in the cytosol, excluding the nucleus, as markers of translocation. These values (*F*) were normalized to each initial value (*F*_*0*_), and the relative fluorescence change was referred to as *F/F*_*0*_. The cells expressing GFP-tagged proteins were loaded with 2 μM Fura2 for the measurement of intracellular Ca^2+^ concentration [Ca^2+^]_*i*_ in the standard extracellular solution for 30 min at room temperature. The cells were washed twice and used within 2 h. Fura2 was excited at wavelengths alternating between 340 and 380 nm, and emissions were collected using the same bandpass filter used for the GFP fluorescence. A shortpass filter of 330–495 nm was used to reduce the background fluorescence between the dichroic mirror and the emission filter, which allowed for simultaneous measurements of GFP and Fura2 fluorescence. We previously determined that GFP and Ca^2+^ signals were distinguishable under these experimental conditions [26].

#### Total internal reflection fluorescence microscopy, or evanescent wave microscopy

To obtain a high signal-to-noise ratio as compared with conventional epifluorescence microscopy, we installed a total internal reflection fluorescence microscopy (TIRFM) unit (Olympus Corp., Tokyo, Japan) on an Olympus inverted microscope (60x/1.45 numerical aperture oil immersion objective) that was equipped with an automatic focus device (ZDC2) and a digital complementary metal oxide semiconductor (CMOS) camera (ORCA-Flash4.0, C11440, Hamamatsu Photonics, Hamamatsu, Japan). Incidental light was introduced from the objective lens for TIRFM to generate the electromagnetic zone, or the so-called “evanescent field.” The evanescent wave selectively excites fluorophores within 100 nm of the glass–water interface, which enabled us to monitor fluorescent proteins at and beneath the plasma membrane of a cell. GFP was excited by a 488-nm laser with a 1.3 neutral density filter (Edmond Optics, Tokyo, Japan), and emissions were collected through a 520/35-nm bandpass filter (Semrock, Rochester, NY, USA). HCI_MAGE_ software (Hamamatsu Photonics) was used to capture fluorescence images. The fluorescence intensity of a range of interest (ROI) in individual cells was measured and analyzed on an Aquacosmos imaging station (Hamamatsu Photonics).

### Measurement of insulin secretion

Insulin secretion from INS-1D cells was measured in a static incubation system as described previously [33]. INS-1 cells were subcultured in 35-mm dishes and grown to 80–90% confluence for 3–4 days. INS-1 cells were preincubated in KRB buffer containing 3 mM glucose at 37°C in a humidified incubator for 1 h. The solution was then replaced with KRB alone or KRB containing various test agents. Antennapedia, antp-PKCα, and antp-PKCε were added 1 h prior to the insulin secretion experiment. The stimulation time was carefully adjusted to standardize the time required for solution changes and sample collection. The experiments were terminated by withdrawing the supernatant solution after 1 h of incubation. The supernatant was then placed in an ice bath. Samples were kept at −20°C until further analysis. Insulin concentration was measured using an insulin enzyme-linked immunosorbent assay kit (Morinaga Institute of Biological Science, Kanagawa, Japan). All samples were assayed in triplicate.

### Statistical analysis

Data are given as means ± standard error. Statistical significance was evaluated using student’s t-test for paired observations. Multiple comparisons were examined by one-way analysis of variance with post hoc Fisher’s LSD test. A *p* value < 0.05 was considered to be statistically significant. Data were analyzed using BellCurve for Excel (Social Survey Research Information Co., Ltd., Tokyo, Japan).

## Results

### GW9508 enhances glucose-stimulated insulin secretion from INS-1D cells

**Fig 1:**
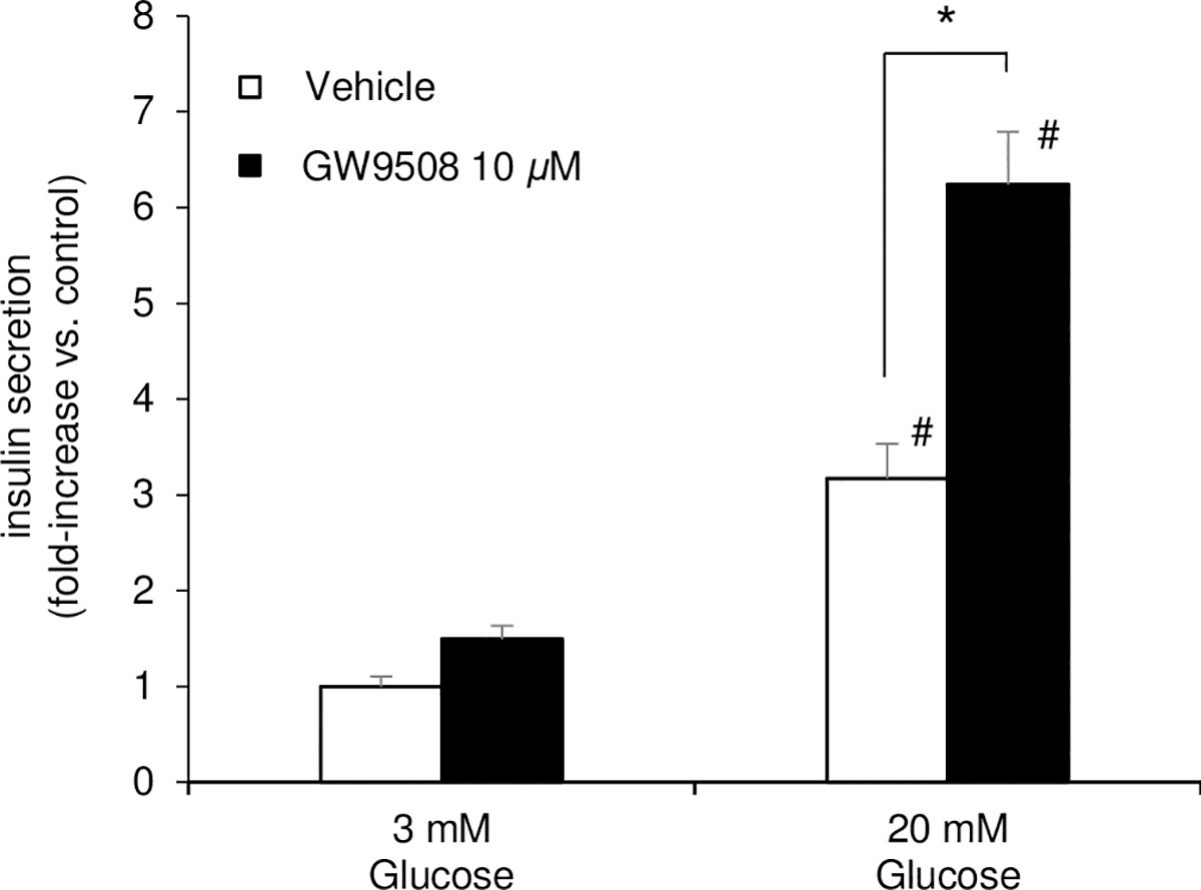
GW9508 enhances insulin secretion only at 20 mM glucose in INS-1D cells. INS-1D cells were incubated for 1 h with Krebs-Ringer buffer (KRB) containing 3 mM or 20 mM glucose in the presence or absence of GW9508, an agonist of G-protein coupled receptor 40 (GPR40). Data shown are mean ± standard error of the mean of three independent experiments with triplicate samples in each group. *p < 0.05 vs. absence of GW9508; #*p* < 0.05 vs. 3 mM glucose.

First, we examined GSIS from INS-1D cells in the presence or absence of GW9508. As expected, 10 μM GW9508 enhanced insulin secretion at a stimulatory concentration (20 mM) of glucose (Fig 1). At the substimulatory concentration (3 mM) of glucose, GW9508 did not significantly increase insulin secretion (Fig 1).

### GW9508 translocates MARCKS-GFP from the plasma membrane to the cytosol at a substimulatory concentration of glucose

**Fig 2:**
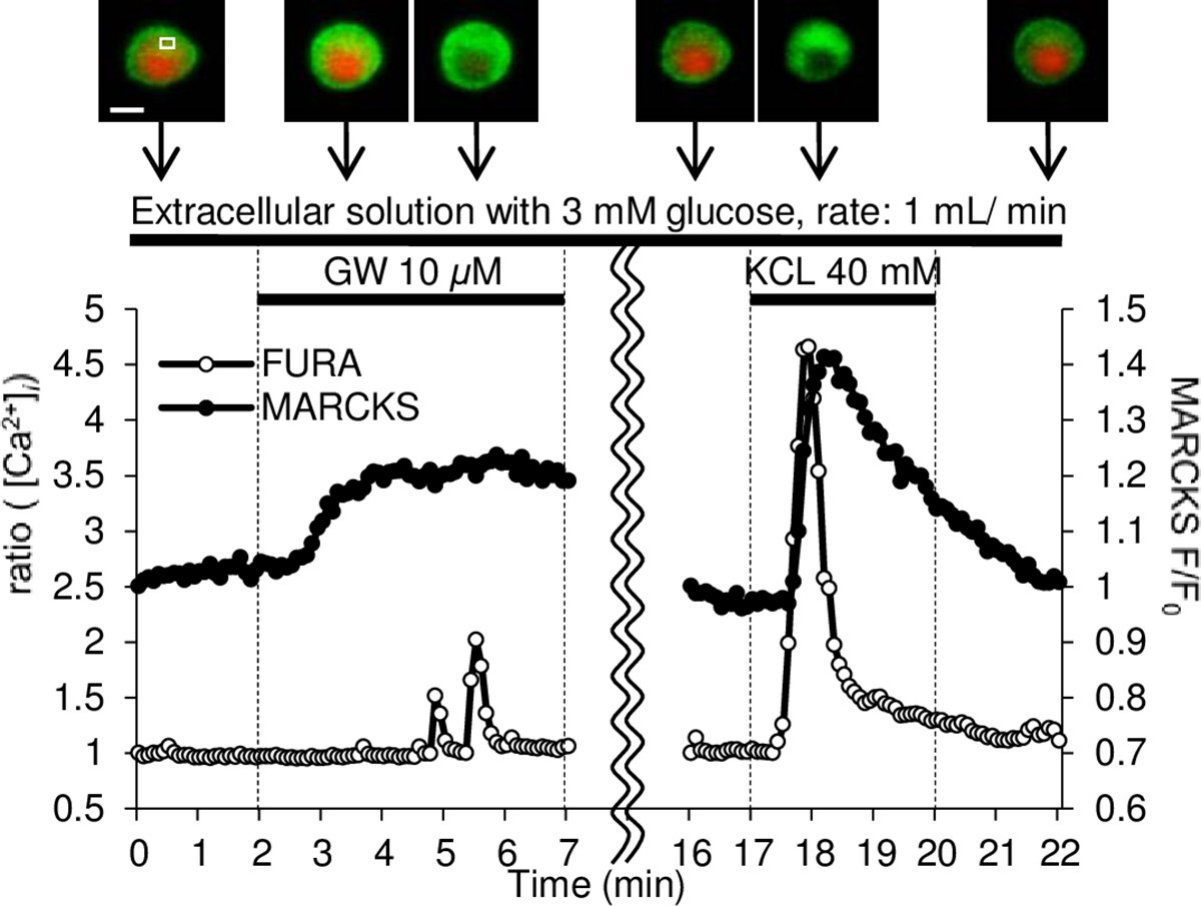
GW9508 induces sustained MARCKS-GFP translocation at 3 mM glucose. Experiments were performed using epifluorescence microscopy. Simultaneous monitoring of green fluorescent protein (GFP)-tagged myristoylated alanine-rich C kinase substrate (MARCKS-GFP; filled circles) translocation (as a marker of PKC activation) and intracellular Ca^2+^ concentration ([Ca^2+^]_*i*_) (open circles). The intensity of Fura2 for [Ca^2+^]_*i*_ measurement is represented as the 340/380 nm ratio. These values and the fluorescence intensity (*F)* of MARCKS-GFP were normalized to each initial value (*F*_*0*_; ratio [Ca^2+^]_*i*_ and MARCKS *F/F*_*0*_). INS-1D cells were perfused with extracellular solution containing 3 mM glucose at 1 mL per minute from 5 minutes before the start of the experiment to the end. To avoid light-induced cell damage, monitoring was paused from 7 to 16 minutes after starting the experiment. At 16 minutes, the distribution of MARCKS-GFP in cells was similar to that observed just after starting the experiment. Images were taken at the times indicated by the arrows. The bar represents 10 μm. The regions of interest in the cytosol are indicated by a white box. When MARCKS-GFP (green) was translocated from the plasma membrane to the cytosol, MARCKS *F/F*_*0*_ in the cytosol increased. When the fluorescence intensity at 380 nm (red) declined, [Ca^2+^]_*i*_, represented as the 340/380 nm ratio, increased (eight independent experiments, 81 cells).

We used GFP-tagged MARCKS, a putative substrate for PKC [34], as a marker of PKC activation to examine the mechanism of GW9508 activation of PKC in living cells. When activated PKC phosphorylates plasma membrane-anchored MARCKS, the phosphorylated MARCKS is translocated from the plasma membrane to the cytosol [35]. This translocation can be identified by reciprocal changes in the fluorescence intensity of MARCKS-GFP between the cytosol and the plasma membrane [26]. Thus, we measured the relative fluorescence change in MARCKS-GFP in the cytosol. MARCKS translocation and [Ca^2+^]_*i*_ levels in INS-1 cells stably expressing MARCKS-GFP were monitored simultaneously. To reduce the effects of glucose on GW9508-induced signal transduction as much as possible, we used a standard extracellular solution containing 3 mM glucose, which is substimulatory in terms of electrical activity and insulin secretion. These conditions were used to evaluate PKC activation by GW9508 in real time; Fig 2 shows a representative experiment (n = 81).

At 10 μM GW9508, we noted sustained translocation of MARCKS-GFP to the cytosol (Fig 2). In contrast to the transient MARCKS-GFP translocation that followed a change in [Ca^2+^]_*i*_ induced by a depolarizing concentration of potassium (40 mM KCL), GW9508-induced translocation of MARCKS-GFP was not affected by changes in [Ca^2+^]_*i*_, indicating that GW9508 activated PKC in a Ca^2+^-independent manner (Fig 2).

### Stimulatory glucose concentration changes GW9508-induced translocation of MARCKS-GFP from a Ca^2+^-independent to a Ca^2+^-dependent mechanism

**Fig 3:**
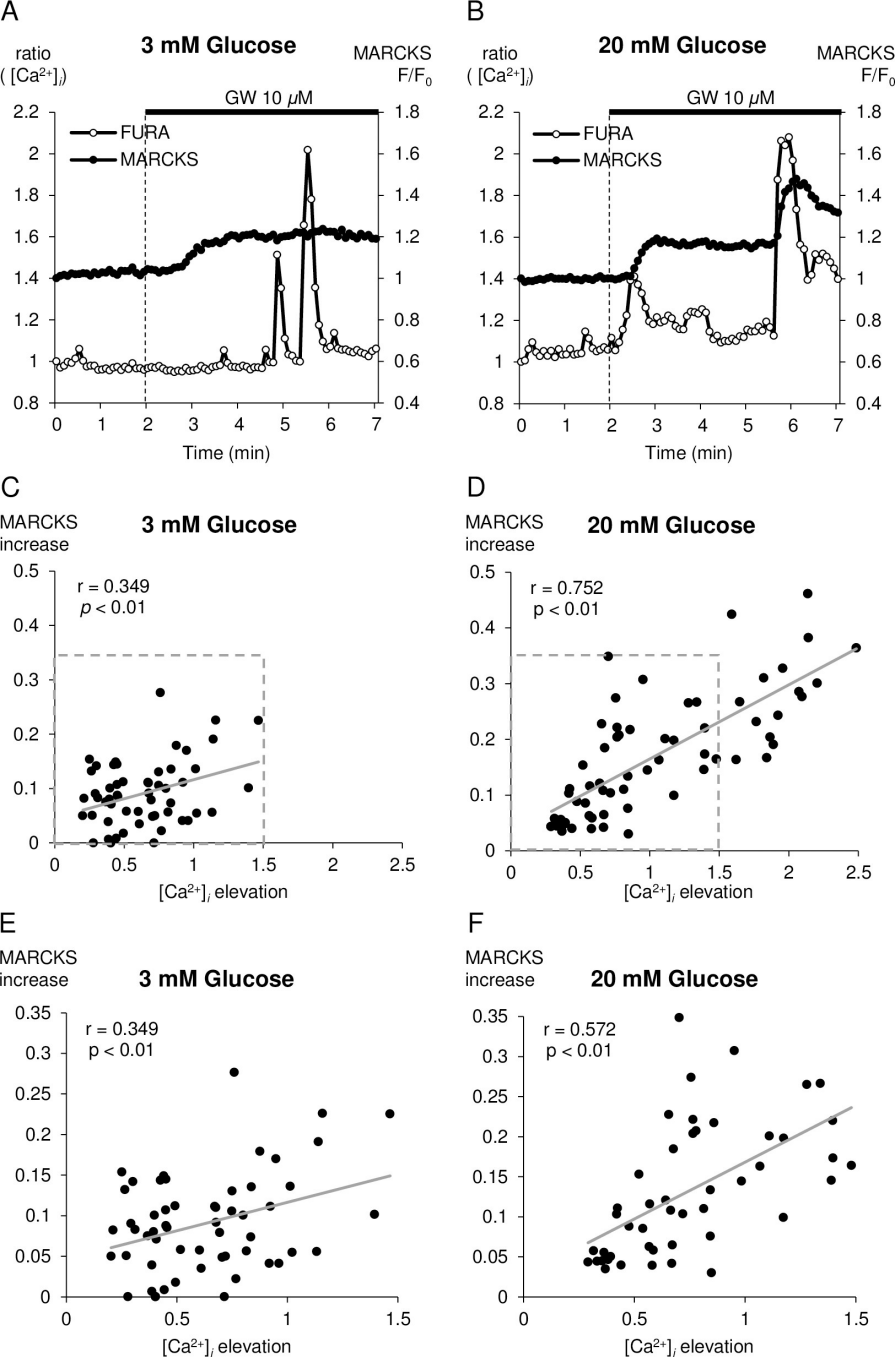
GW9508 evokes transient translocation of MARCKS-GFP following Ca^2+^ oscillations at 20 mM glucose. (A, B): Representative data showing the translocation of green fluorescent protein (GFP)-tagged myristoylated alanine-rich C kinase substrate (MARCKS-GFP) and intracellular Ca^2+^ concentration [Ca^2+^]_*i*_ under stimulation by GW9508 in an extracellular solution containing (A) 3 mM glucose (the same data as in Fig 2 but with different scales; eight independent experiments, 81 cells) or (B) 20 mM glucose (four independent experiments, 80 cells). Biphasic translocation of MARCKS-GFP occurred after the second sharp increase in Ca^2+^ at 20 mM glucose. Scatterplots of the ratio of [Ca^2+^]_*i*_-related increases in the relative fluorescence intensity of MARCKS-GFP in the cytosol *versus* the elevation in ratio [Ca^2+^]_*i*_ during GW9508 application at (C) 3 mM glucose (eight independent experiments, 56 cells) or (D) 20 mM glucose (four independent experiments, 63 cells). Cells that did not respond to GW9508 were excluded from the calculation. To count the cells that responded to GW9508, only cells that were almost unchanged in fluorescence of MARCKS-GFP before the application of GW9508 were chosen for study at 3 mM glucose. (E, F) To equalize the [Ca^2+^]_*i*_ elevation, only cells with an [Ca^2+^]_*i*_ ratio elevated by 1.5 were selected for analysis from C (all 56 cells) and D (47 cells) (dashed box in C and D).

We demonstrated that GW9508 increased GSIS and did not amplify insulin secretion at 3 mM glucose (Fig 1). Next, we compared GW9508-induced MARCKS translocation at 3 mM and 20 mM glucose. The application of GW9508 resulted in sustained translocation of MARCKS-GFP to the cytosol, as well as multiple transient translocations of MARCKS-GFP that occurred just following sharp increases in [Ca^2+^]_*i*_, at 20 mM glucose (Fig 3B). We then plotted the [Ca^2+^]_*i*_-related increases in the *F/F*_*0*_ of MARCKS-GFP in the cytosol against sharp elevations in [Ca^2+^]_*i*_ during the 5-min application of GW9508 at 3 mM or 20 mM glucose. The correlation between the increase in MARCKS and [Ca^2+^]_*i*_ elevation was weak at 3 mM glucose (r = 0.349; *p* < 0.01), but stronger at 20 mM glucose (r = 0.752; *p* < 0.01) (Fig 3C and 3D). A strong correlation between [Ca^2+^]_*i*_ elevation and the increase in MARCKS also existed below a 1.5 elevation of the [Ca^2+^]_*i*_ ratio at 20 mM glucose (r = 0.572; *p* < 0.01) (Fig 3E and 3F). These observations suggest that GW9508-evoked Ca^2+^ signals induced the activation of PKC more robustly at a stimulatory concentration of glucose.

### Profiles of PKCα and PKCε translocation in response to GW9508 at substimulatory and stimulatory concentrations of glucose

**Fig 4:**
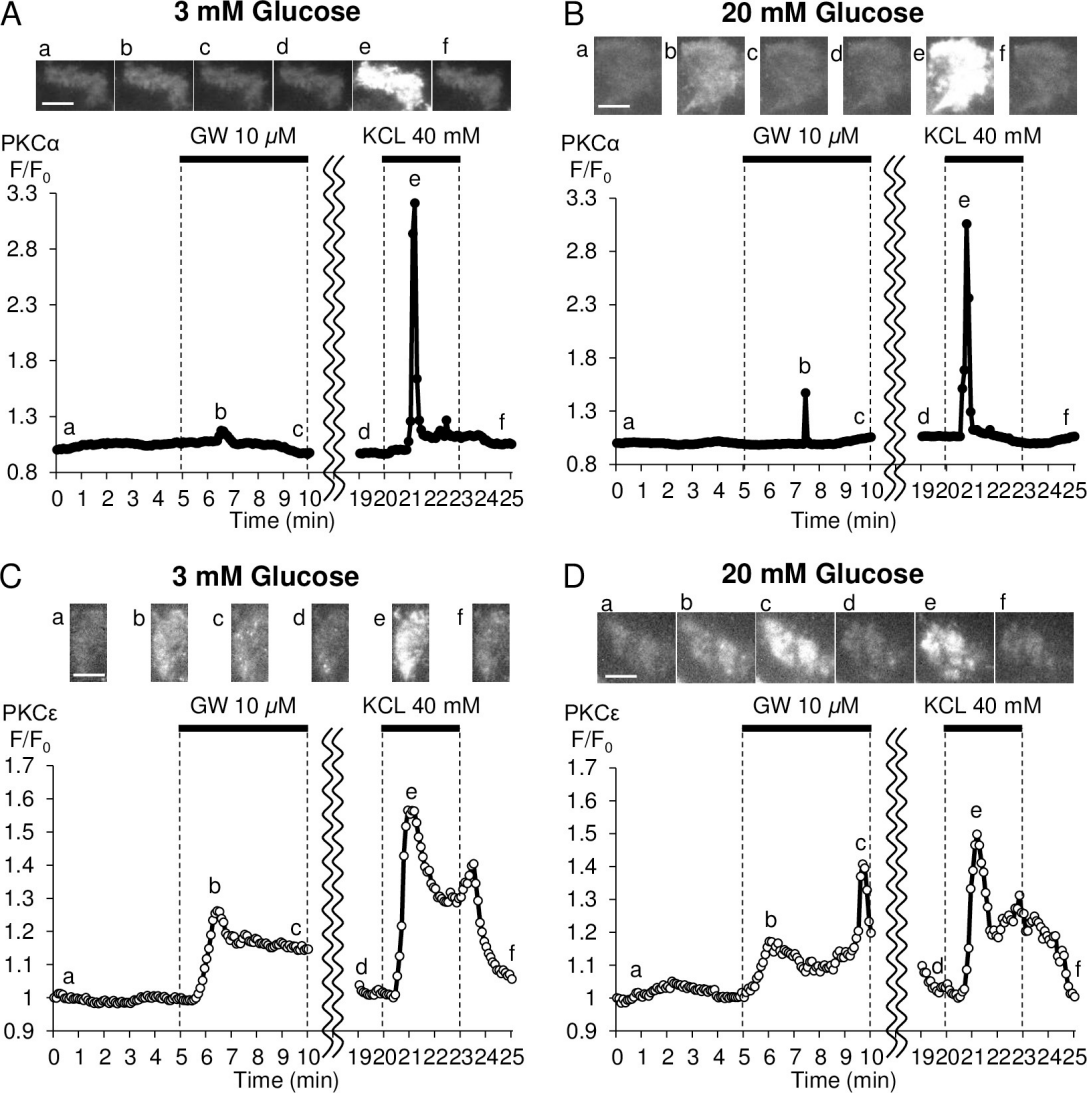
Temporal profile of PKC*α* and PKC*ε* translocation at 3 or 20 mM using total internal reflection fluorescence microscopy. Representative data of the relative change in fluorescence intensity (*F/F*_*0*_) of PKC*α*-GFP (A, B) or PKCε-GFP (C, D) at the plasma membrane. Images a-f were taken at the times indicated in each graph. Scale bars = 10 μm. When green fluorescent protein (GFP)-tagged PKC*α* or PKC*ε* is translocated from the cytosol to the plasma membrane, *F/F*_*0*_ increases. (A, B) Translocation of PKC*α*-GFP evoked by GW9508 at 20 mM glucose (B; 15 independent experiments, 165 cells) was higher than that at 3 mM glucose (A; 19 independent experiments, 283 cells), but lower than that induced by 40 mM KCL. (C, D) Translocation of PKC*ε*-GFP induced by GW9508 at 20 mM glucose (D; 15 independent experiments, 57 cells) was biphasic, higher than that at 3 mM glucose (C; 18 independent experiments, 47 cells), and comparable with that induced by 40 mM KCL.

The observations above prompted us to investigate whether there were differences in the activation of PKC isoforms between substimulatory and stimulatory concentrations of glucose. We examined the GW9508-evoked translocation of PKC*α*-GFP and PKC*ε*-GFP in transfected INS-1D cells using TIRFM. Only 17% of experimental cells showed transient translocation of PKC*α* in response to GW9508 at 3 mM glucose (Fig 4A, Table 1). At 20 mM glucose, more than twice the number of cells responded to GW9508, i.e. 35% of cells showed transient PKC*α* translocation (Fig 4B, Table 1). Conversely, 51% of cells showed transient or sustained translocation of PKC*ε* at 3 mM glucose (Fig 4C, Table 1). Interestingly, in addition to sustained translocation of PKC*ε*, GW9508 also elicited transient translocation of PKC*ε* from a higher percentage of cells at 20 mM (51%) compared with 3 mM (20%) glucose (*p* < 0.01) (Fig 4D, Table 1). However, the response time for translocation of both PKC*α* and PKC*ε* did not differ significantly between 3 mM glucose and 20 mM glucose (Table 1). These results suggest that PKC*ε* played a dominant role in GW9508-induced MARCKS activation at substimulatory and stimulatory concentrations of glucose.

**Table 1:** Effect of GW9508 on PKC*α* and PKC*ε* activation at 3 mM and 20 mM glucose.

	3 mM Glucose	20 mM Glucose
**PKC*α***		
**Fraction of INS-1 cells responding to GW9508**	47 of 283 (17%)	58 of 165 (35%)*
**Lag time (sec.)**	131.4 ± 11.9	141.8 ± 10.7
**PKC*ε***		
**Fraction of INS-1 cells responding to GW9508**	25 of 49 (51%)	38 of 57 (67%)
**Fraction of cells with a transient response**	10 of 49 (20%)	29 of 57 (51%)*
**Fraction of cells with a sustained response**	21 of 49 (43%)	28 of 57 (49%)
**Lag time (sec.)**	56.6 ± 5.3	77.6 ± 7.8

Only cells exhibiting an increase in the *F/F*_*0*_ of ≥ 0.1 over the baseline level were counted as responding to GW9508. The response to GW9508 was further categorized into cell fractions with a sustained or transient translocation of PKC. Lag time = response time of PKC, and is shown as mean ± standard error of the mean.

*p < 0.01 vs. GW9508 at 3 mM glucose.

### Effect of PKC inhibitors on GW9508-potentiated insulin secretion in INS-1 cells

**Fig 5:**
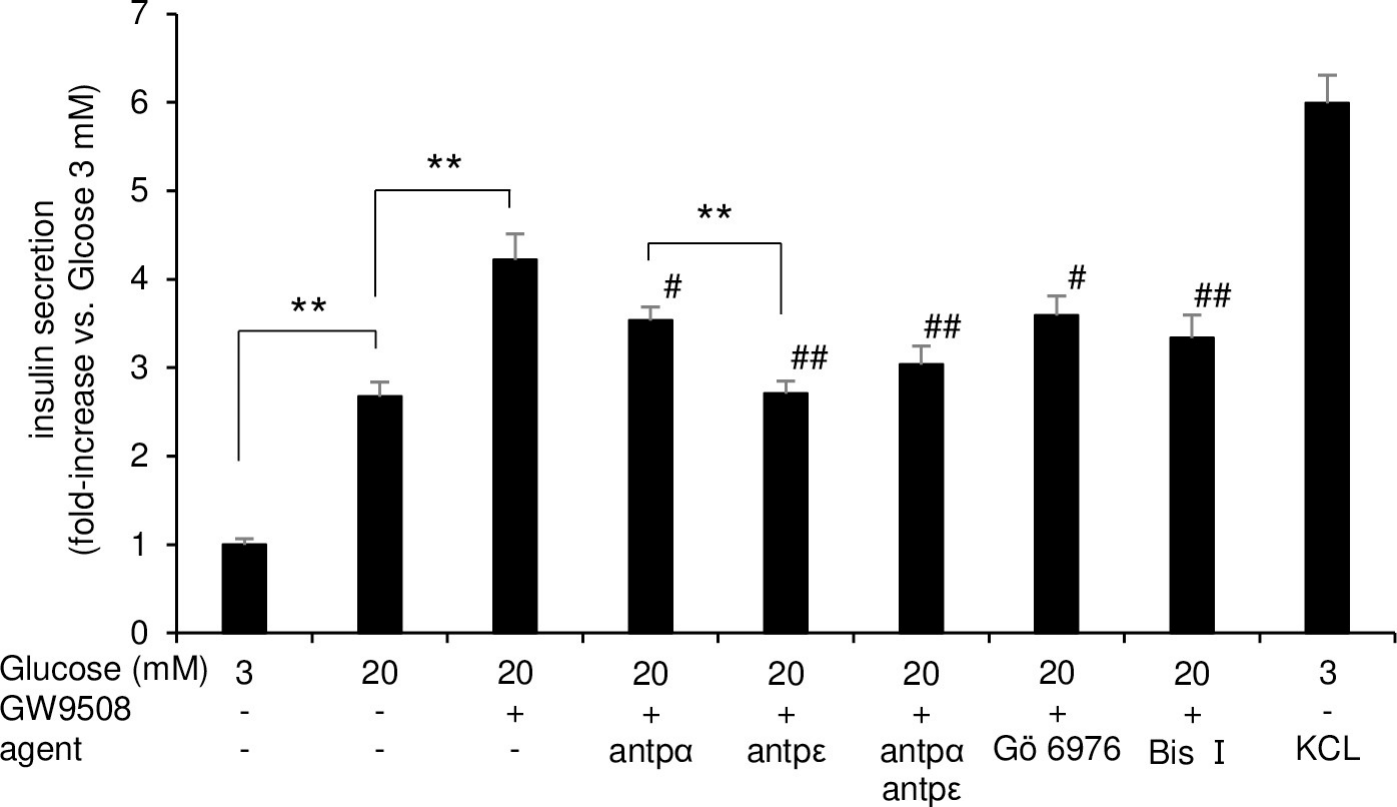
PKC inhibitors suppress GW9508-induced insulin secretion in INS-1D cells. INS-1D cells were incubated for 1 h in Krebs-Ringer buffer containing 3 mM or 20 mM glucose with stimulation by GW9508 in the absence or presence of 75 μM antennapedia (antp), 75 μM antp-PKC*α* (antp*α*), 75 μM antp-PKC*ε* (antp*ε*), both antp*α* and antp*ε*, 1 μM Gö 6976, or 1 μM bisindolylmaleimide I (BIS I). Data are shown as mean ± standard error of the mean of 12 independent experiments with triplicate samples in each group. *p < 0.05; **p < 0.01; #*p* < 0.05 vs. GW9508 at 20 mM glucose; ##*p* < 0.01 vs. GW9508 at 20 mM glucose.

We tested the isoform-specific roles of the two PKCs in GW9508-potentiated insulin secretion in INS-1D cells using antp-PKC*α* and antp-PKC*ε*. GW9508-induced insulin secretion at 20 mM glucose was significantly reduced by 75 μM antp-PKC*α* (*p* < 0.05) and 75 μM antp-PKC*ε* (*p* < 0.01) (Fig 5). Antp-PKC*ε* inhibited insulin secretion more potently than antp-PKC*α* at 20 mM glucose (*p* < 0.05) (Fig 5). Double inhibition with antp-PKC*α* and *ε* did not decrease insulin secretion below the level inhibited by antp-PKC*ε* alone (Fig 5). To strengthen these results, we also tested Gö 6976, an inhibitor of conventional PKC, and BIS I, a broad PKC inhibitor. While both Gö 6976 and BIS I significantly reduced GW9508-induced insulin secretion, BIS I had a stronger effect (Fig 5). These results agreed with those using antp-PKC*α* and antp-PKC*ε*. Taken together, the results of the PKC inhibitor experiments suggest that both PKC isoforms, but PKC*ε* in particular, were responsible for GW9508-potentiated insulin secretion in INS-1D cells.

## Discussion

We demonstrated in this study that: 1) GW9508-induced activation of PKC was mainly characterized by sustained PKC*ε*, which was inconsistent with changes in [Ca^2+^]_*i*_ at a substimulatory concentration of glucose (Figs 2, 3A, 3C, 4A and 4C); 2) a stimulatory concentration of glucose enabled GW9508-induced transient translocation of PKC*α* and PKC*ε* that followed changes in [Ca^2+^]_*i*_ (Figs 3B, 3D, 4B and 4D); and 3) GW9508-potentiated GSIS was disrupted by two PKC inhibitory peptides, with more marked inhibition of PKC*ε*, in INS-1D cells (Fig 5). These results indicate that PKC*ε* is directly activated by GW9508, independent of glucose concentration, and suggest that the shift in PKC*α* and PKC*ε* activation from a stable, sustained mode to a transient mode is involved in the potentiation of GSIS.

In a previous report, we demonstrated that Ca^2+^ influx via voltage-dependent Ca^2+^ channels (VDCCs) can activate PKC [26], and that GLP-1-induced PKC activation is transient and Ca^2+^-dependent [7]. Nevertheless, in this study PKC activation by GW9508 was independent of elevations in [Ca^2+^]_*i*_ and sustained during the application of GW9508 at a substimulatory concentration of glucose (Figs 2 and 3A). GPR40 signaling generates IP_3_ and DAG via PLC activation [13]. IP_3_ induces Ca^2+^ release from the ER, and DAG activates PKC directly [14,36]. Thus, GPR40-mediated DAG, but not IP_3_, could play a key role in sustained PKC activation that is induced by GW9508 at a substimulatory concentration of glucose. However, a significant but weak correlation between PKC activation and [Ca^2+^]_*i*_ elevation was confirmed, which could represent a population of INS-1 cells that allowed Ca^2+^ influx through VDCCs (Fig 3C and 3E). The TIRFM imaging experiments showed that GW9508 induced sustained PKC*ε* activation, but not sustained PKC*α* activation, at a substimulatory concentration of glucose (Fig 4A and 4C). These results suggest that IP_3_-induced Ca^2+^ release was insufficient for activation of the conventional PKC*α* isotype in response to GW9508 at a substimulatory concentration of glucose in INS-1D cells, whereas DAG was sufficient for activation of the novel PKC*ε* isotype.

We have shown here that a stimulatory concentration of glucose altered GW9508-induced PKC activation from a Ca^2+^-independent to a Ca^2+^-dependent mechanism, despite the amplitudes of induced [Ca^2+^]_*i*_ elevations over the entire cell being similar between the stimulatory and substimulatory concentrations of glucose (Fig 3A–3F). On the other hand, nifedipine, a blocking agent of VDCCs, attenuated the transient translocation of MARCKS-GFP that was induced by GW9508 at a stimulatory concentration of glucose (S1 Fig). The TIRFM imaging experiments showed that GW9508 increased the fraction of cells that underwent transient PKC*α* activation at a stimulatory concentration of glucose (Fig 4B, Table 1). High concentrations of glucose are known to stimulate insulin secretion through an intracellular pathway involving an increase in the intracellular adenosine triphosphate (ATP)/adenosine diphosphate (ADP) ratio and closure of K_ATP_ channels, followed by membrane depolarization, which leads to the activation of VDCCs and a rise in [Ca^2+^]_*i*_ [37–40]. In our previous report, we demonstrated that Ca^2+^ influx was a much stronger stimulus of PKC*α* translocation than Ca^2+^ mobilization from intracellular stores in INS-1D cells [26]. In light of that result, it could be interpreted that the Ca^2+^ mobilization that was induced by GW9508-generated IP_3_ in the current study failed to translocate PKC*α* at the substimulatory concentration of glucose, and that K_ATP_-induced Ca^2+^ influx through VDCCs activated PKC*α* at the stimulatory concentration of glucose. However, INS-1D cells are known to exhibit a strong electrical and insulin response to KCl stimulation and a less potent response to glucose stimulation. This could explain the lack of differentiation in the response time of PKC*α* between the substimulatory and stimulatory concentrations of glucose (Table 1).

TIRFM imaging also showed that GW9508 induced transient activation of PKC*ε*, in addition to the sustained activation, at a stimulatory concentration of glucose (Fig 4D, Table 1). We demonstrated in a previous report that Ca^2+^ influx via VDCCs could activate PLC [26]. Among all known PLC isoforms, the PLC*δ* isoforms are the most sensitive to Ca^2+^ [41]. Thus, Ca^2+^ influx via VDCCs would be expected to activate PLC, and PLC*δ*, in particular, leading to the transient activation of PKC*ε* that we observed at the stimulatory concentration of glucose. Another recent report concluded that the rapid activation of PKC*ε* in the plasma membrane is due to exocytotic release of ATP, with autocrine feedback activation of P2Y_1_ purinoceptors, which in turn induces DAG via PLC activation [42,43]. Thus, glucose-stimulated Ca^2+^ signaling and autocrine signaling could be sufficient to trigger the rapid activation of PLC, which activates PKC*ε* by generating DAG.

The exact mechanism of GPR40-potentiated GSIS in *β*-cells remains unclear. Here, we observed that both PKC*α* and, to a greater degree, PKC*ε*, were involved in GW9508-potentiated insulin secretion, but only at a stimulatory concentration of glucose (Figs 1 and 5). However, 12-*O*-tetradecanoylphorbol 13-acetate (TPA), which binds the diacylglycerol site to potently activate PKC, has been reported to induce insulin secretion at substimulatory as well as stimulatory concentrations of glucose in insulin-producing cells [44–46]. This discrepancy could be explained by a requirement for PKC activation to exceed a threshold value for GPR40-mediated insulin secretion to occur. We also observed a larger amplitude of [Ca^2+^]_*i*_ increase in GW9508-mediated INS-1D cells compared with vehicle at a stimulatory concentration of glucose (S2 Fig). A recent report showed that fasiglifam, another GPR40 agonist, enhanced GSIS through both IP_3_-mediated amplification of Ca^2+^ oscillations and DAG-mediated augmentation of downstream secretory mechanisms independent of Ca^2+^ oscillations [47]. Thus, IP_3_ might be involved in insulin secretion only at a stimulatory concentration of glucose, as shown in this study. Recently, it was reported that GPR40 depolarizes the plasma membrane and increases background current via the transient receptor potential canonical 3 (TRPC3) channel at a substimulatory concentration of glucose in pancreatic *β*-cells [48]. TRPC3 is a class of nonselective cation channels that is activated by PLC/PKC signaling, not Ca^2+^ from the ER, resulting in the potentiation of GSIS [48]. It has also been reported that physiological concentrations of GLP-1 stimulate insulin secretion through the PKC-dependent activation of transient receptor potential melastatin 4 (TRPM4) and TRPM5, which are Na^+^-permeable cation channels [8]. Thus, the GW9508-induced PKC*ε* activation at substimulatory concentrations of glucose that we observed here might have involved TRPC3 and TRPM activation, which potentiate Ca^2+^ influx at stimulatory concentrations of glucose.

PKC*ε* played a dominant role over that of PKC*α* in GW9508-induced insulin secretion by INS-1D cells (Fig 5), consistent with evidence of the dominant translocation of PKC*ε* induced by GW9508 (Fig 4A–4D). PKC*ε* is involved in GSIS, and several studies have shown that the inhibition of the function of PKC*ε* is associated with reduced GSIS [24,25,49]. Activated PKC*ε* has been shown to localize to insulin granules, enhance biosynthetic pathways of proinsulin, and induce the processing of proinsulin to mature insulin [24,49]. Another recent study showed that novel PKCs stimulated mitochondrial ATP production via ERK1/2 signaling [50], which increased the cytosolic ATP/ADP ratio [51]. These mechanisms may contribute to the GW9508-enhanced insulin response to a stimulatory concentration of glucose via PKC*ε* activation, as observed in our study. In contrast, the contribution of PKC*α* activation to GSIS remains a subject of debate. Inconsistencies in the data may be explained in part by the different effects of PKC*α* on the initial and late phases of secretion [52].

We also investigated the effect of *γ*-linolenic acid (*γ*-LA), a natural ligand of GPR40, and found that it elicited insulin secretion not only at the stimulatory but also at the substimulatory concentration of glucose (S5 Fig). At a substimulatory concentration of glucose, *γ*-LA elicited sustained PKC activation, whereas at the stimulatory concentration of glucose, PKC exhibited not only sustained activation but also transient activation (S3 and S4 Figs, S1 Table). Unlike GW9508, however, *γ*-LA-stimulated insulin secretion was not affected by antp-PKC*α* or antp-PKC*ε* (S5 Fig). These results suggest that receptor-independent pathways rather than the GPR40 pathway are also involved in *γ*-LA–evoked insulin secretion, and could include the malonyl-CoA/long-chain acyl-CoA pathway and triglyceride/free fatty acid cycling via the intracellular metabolism of fatty acids [53]. Our results using GW9508, however, suggest that PKC-dependent pathways are the sole signaling pathways for GPR40-dependent insulin secretion.

In conclusion, the GPR40 agonist GW9508 induced the sustained activation of the novel isoform PKC*ε* at substimulatory concentrations of glucose, and evoked the transient activation of the conventional isoform PKC*α* and PKC*ε* following increases in [Ca^2+^]_*i*_ via VDCCs at stimulatory concentrations of glucose. This activation, which was especially potent for PKC*ε*, was involved in GW9508-potentiated GSIS. GPR40 agonists have the potential to be key drugs for increasing insulin levels with minimal risk of iatrogenic hypoglycemia in patients with type 2 diabetes.

## Supporting information

S1 Fig Nifedipine attenuates GW9508-induced biphasic MARCKS-GFP translocation at 20 mM glucose. INS-1D cells were perfused with extracellular solution with 10 μM nifedipine containing 20 mM glucose at 1 mL per minute from 5 minutes before the start of the experiment to the end. Representative data of translocation of green fluorescent protein (GFP)-tagged myristoylated alanine-rich C kinase substrate (MARCKS-GFP) by GW9508 (four independent experiments, 80 cells).
(TIF)

S2 Fig Effect of GW9508 on intracellular Ca^2+^ level. Elevation of the ratio of the intracellular Ca^2+^ concentration ([Ca^2+^]_*i*_) during GW9508 application at 3 mM (eight independent experiments, 56 cells) or 20 mM glucose (four independent experiments, 63 cells). #*p* < 0.01 vs. GW9508 at 3 mM glucose.
(TIF)

S3 Fig *γ*-linolenic acid induces MARCKS-GFP translocation at 3 mM glucose. Representative epifluorescence microscopy showing the translocation of green fluorescent protein (GFP)-tagged myristoylated alanine-rich C kinase substrate (MARCKS-GFP) by *γ*-linolenic acid (*γ*-LA) (seven independent experiments, 98 cells). INS-1D cells were perfused with extracellular solution containing 3 mM glucose at 1 mL per minute from 5 minutes before the start of the experiment to the end. MARCKS-GFP was monitored at the cytosol. Fluorescence intensity (*F)* values were normalized to the initial value (*F*_*0;*_ MARCKS *F/F*_*0*_). To avoid light-induced cell damage, monitoring was paused from 7 to 16 minutes after the start of each experiment. At 16 minutes, the distribution of MARCKS-GFP in cells was similar to that observed just after starting the experiment.
(TIF)

S4 Fig *γ*-linolenic acid evokes biphasic MARCKS-GFP translocation at 20 mM glucose. Representative epifluorescence microscopy showing the translocation of green fluorescent protein (GFP)-tagged myristoylated alanine-rich C kinase substrate (MARCKS-GFP) by *γ*-linolenic acid (*γ*-LA) in an extracellular solution containing 3 mM glucose (A; the same data as shown in S3 Fig) or 20 mM glucose (B; four independent experiments, 80 cells).
(TIF)

S5 Fig *γ*-linolenic acid-induced insulin secretion and effect of PKC inhibitors in INS-1D cells. INS-1D cells were incubated for 1 h in Krebs-Ringer buffer (KRB) containing 3 mM or 20 mM glucose with stimulation by *γ*-linolenic acid (*γ*-LA) in the presence or absence of 75 μM antennapedia (antp), 75 μM antp-PKC*α*, 75 μM antp-PKC*ε*, or both antp-PKC*α* and antp-PKC*ε*. Data are shown as mean ± standard error of the mean of three independent experiments with triplicate samples in each group. *p < 0.05; **p < 0.01.
(TIF)

S1 Table Effect of *γ*-linolenic acid on PKC activation at 3 mM and 20 mM glucose. The response to GW9508 was further categorized into cell fractions with a sustained or transient translocation of green fluorescent protein (GFP)-tagged myristoylated alanine-rich C kinase substrate (MARCKS-GFP). Lag time = the response time of MARCKS-GFP, and is shown as mean ± standard error of the mean. *p < 0.05 vs. GW9508 at 3 mM glucose; **p < 0.01 vs. GW9508 at 3 mM glucose.
(DOCX)
