# Supplementary figures and images for: G-protein-coupled receptor 40 agonist GW9508 potentiates glucose-stimulated insulin secretion through activation of protein kinase Cα and ε in INS-1 cells
Source: PLoS One. 2019 Sep 9;14(9):e0222179. doi: 10.1371/journal.pone.0222179 (PMC6733457; doi:10.1371/journal.pone.0222179)

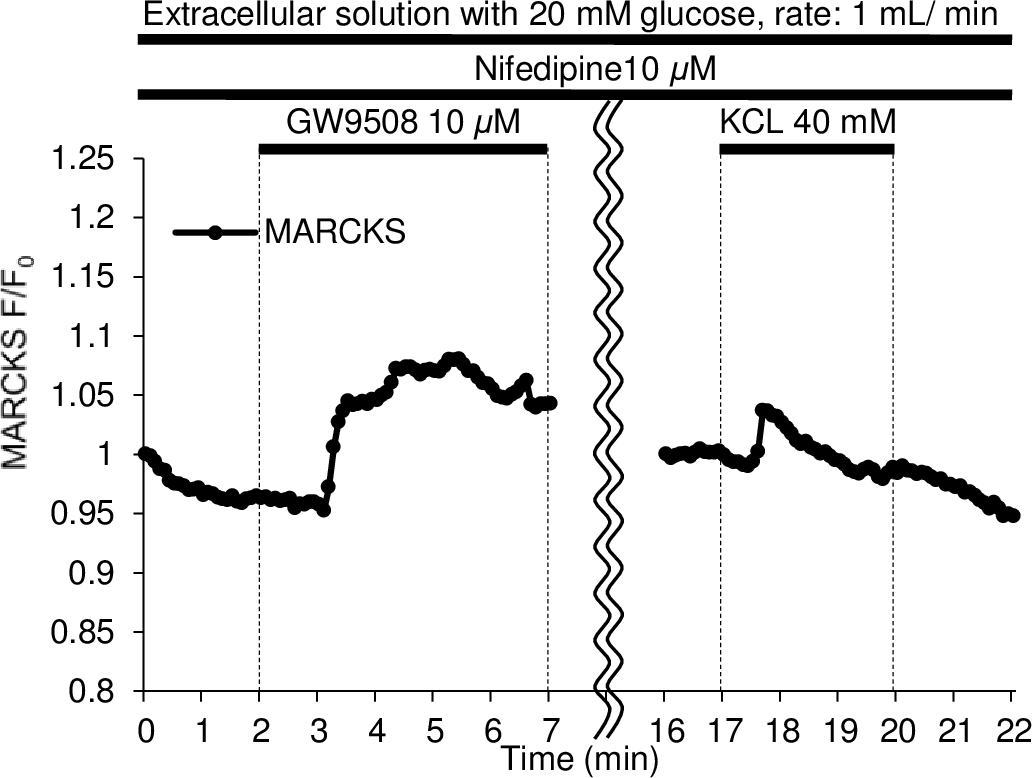

Supplement: S1 Fig — INS-1D cells were perfused with extracellular solution with 10 μM nifedipine containing 20 mM glucose at 1 mL per minute from 5 minutes before the start of the experiment to the end. Representative data of translocation of green fluorescent protein (GFP)-tagged myristoylated alanine-rich C kinase substrate (MARCKS-GFP) by GW9508 (four independent experiments, 80 cells). (TIF) [file pone.0222179.s001.tif]

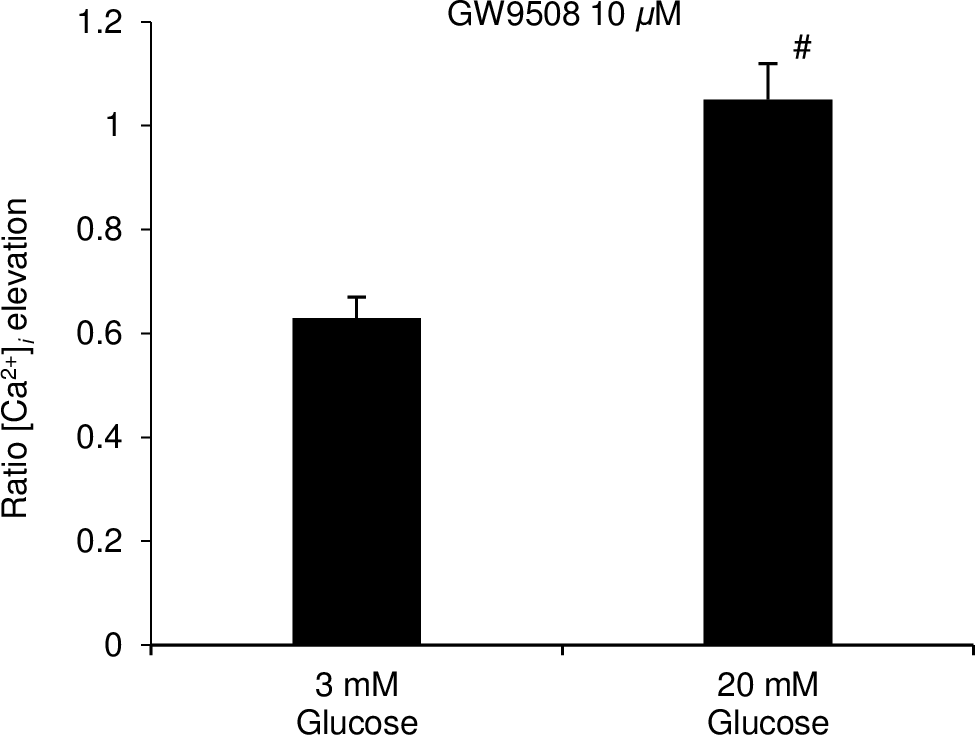

Supplement: S2 Fig — Elevation of the ratio of the intracellular Ca2+ concentration ([Ca2+]i) during GW9508 application at 3 mM (eight independent experiments, 56 cells) or 20 mM glucose (four independent experiments, 63 cells). #p < 0.01 vs. GW9508 at 3 mM glucose. (TIF) [file pone.0222179.s002.tif]

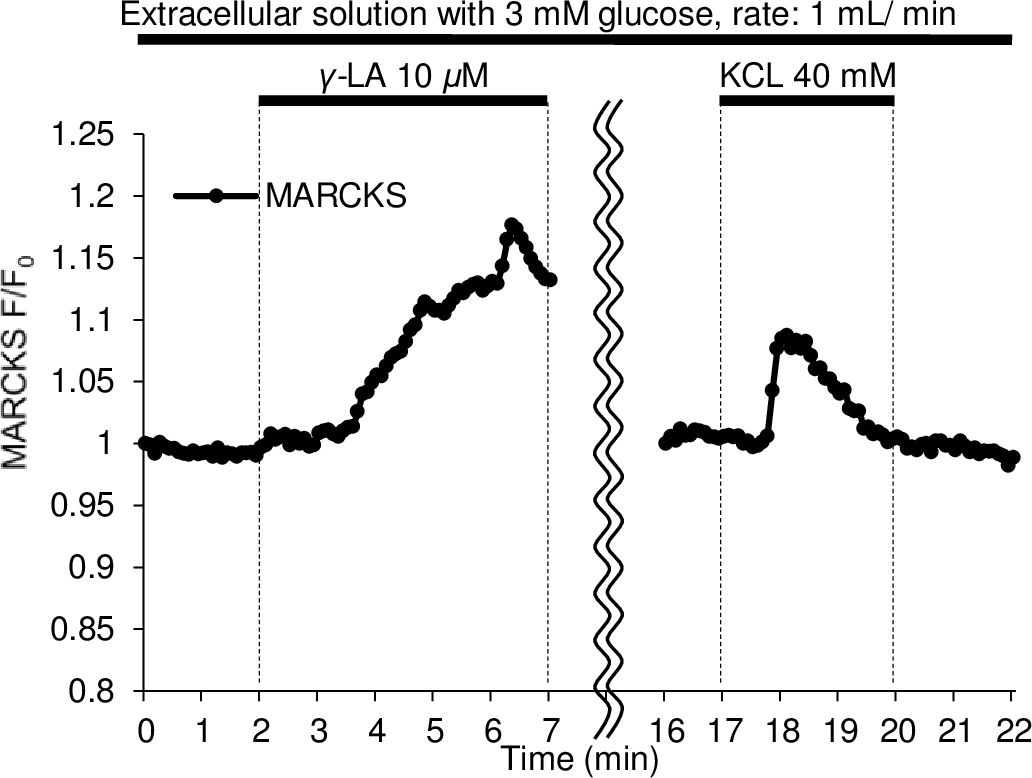

Supplement: S3 Fig — Representative epifluorescence microscopy showing the translocation of green fluorescent protein (GFP)-tagged myristoylated alanine-rich C kinase substrate (MARCKS-GFP) by γ-linolenic acid (γ-LA) (seven independent experiments, 98 cells). INS-1D cells were perfused with extracellular solution containing 3 mM glucose at 1 mL per minute from 5 minutes before the start of the experiment to the end. MARCKS-GFP was monitored at the cytosol. Fluorescence intensity (F) values were normalized to the initial value (F0; MARCKS F/F0). To avoid light-induced cell damage, monitoring was paused from 7 to 16 minutes after the start of each experiment. At 16 minutes, the distribution of MARCKS-GFP in cells was similar to that observed just after starting the experiment. (TIF) [file pone.0222179.s003.tif]

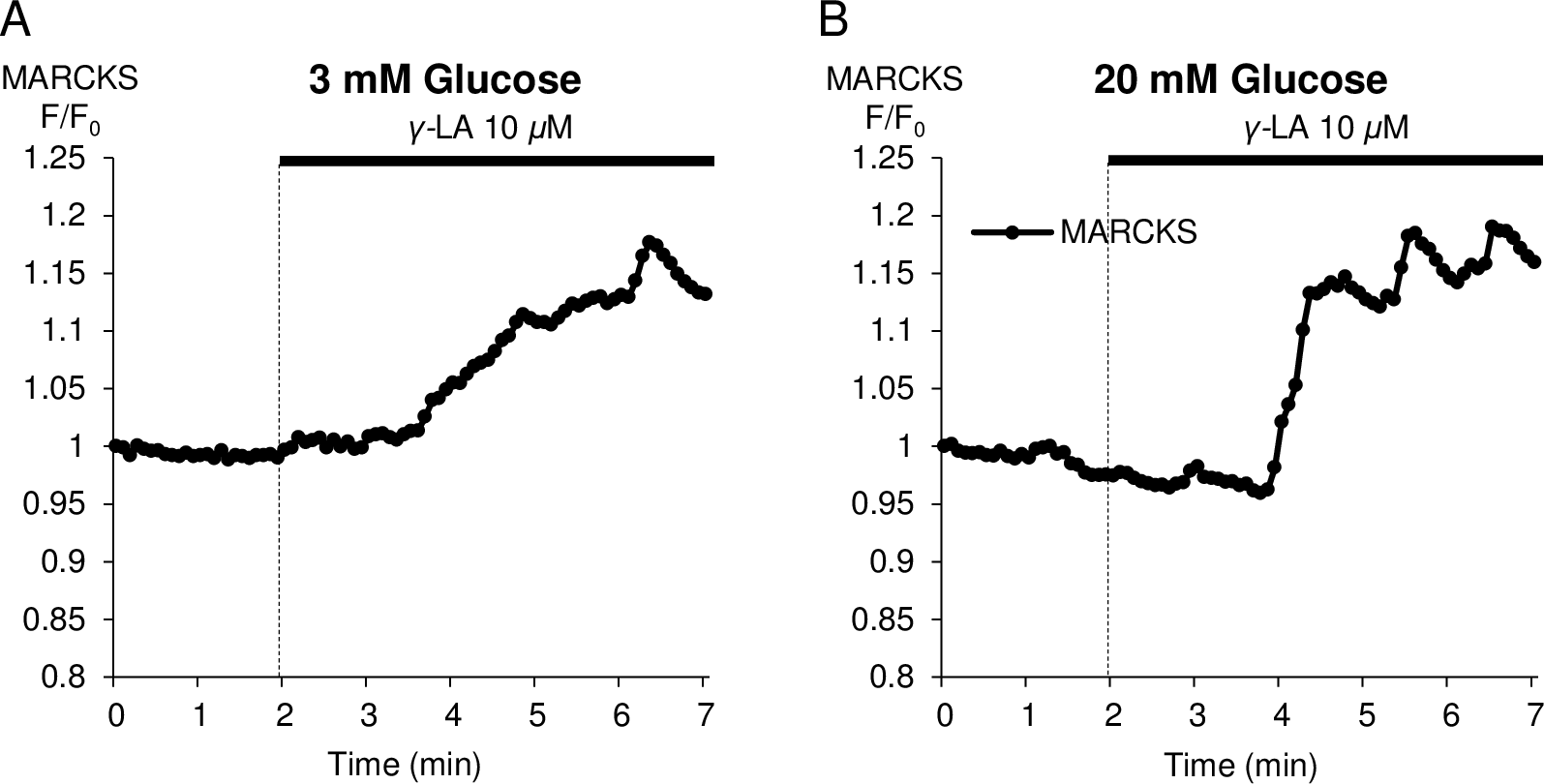

Supplement: S4 Fig — Representative epifluorescence microscopy showing the translocation of green fluorescent protein (GFP)-tagged myristoylated alanine-rich C kinase substrate (MARCKS-GFP) by γ-linolenic acid (γ-LA) in an extracellular solution containing 3 mM glucose (A; the same data as shown in S3 Fig) or 20 mM glucose (B; four independent experiments, 80 cells). (TIF) [file pone.0222179.s004.tif]

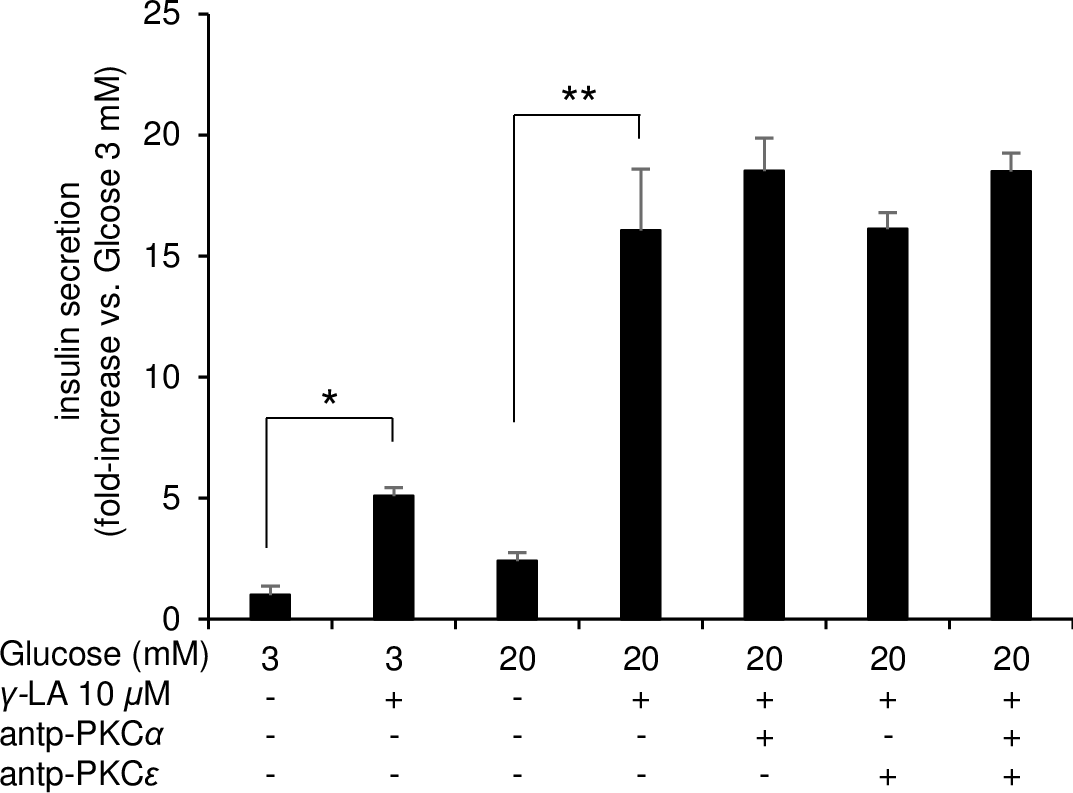

Supplement: S5 Fig — INS-1D cells were incubated for 1 h in Krebs-Ringer buffer (KRB) containing 3 mM or 20 mM glucose with stimulation by γ-linolenic acid (γ-LA) in the presence or absence of 75 μM antennapedia (antp), 75 μM antp-PKCα, 75 μM antp-PKCε, or both antp-PKCα and antp-PKCε. Data are shown as mean ± standard error of the mean of three independent experiments with triplicate samples in each group. *p < 0.05; **p < 0.01. (TIF) [file pone.0222179.s005.tif]
