# Supplementary material for: G-protein-coupled receptor 40 agonist GW9508 potentiates glucose-stimulated insulin secretion through activation of protein kinase Cα and ε in INS-1 cells
Source: PLoS One. 2019 Sep 9;14(9):e0222179. doi: 10.1371/journal.pone.0222179 (PMC6733457; doi:10.1371/journal.pone.0222179)
Supplement: S1 Table — The response to GW9508 was further categorized into cell fractions with a sustained or transient translocation of green fluorescent protein (GFP)-tagged myristoylated alanine-rich C kinase substrate (MARCKS-GFP). Lag time = the response time of MARCKS-GFP, and is shown as mean ± standard error of the mean. *p < 0.05 vs. GW9508 at 3 mM glucose; **p < 0.01 vs. GW9508 at 3 mM glucose. (DOCX) [file pone.0222179.s006.docx]

|  | **3 mM Glucose** | **20 mM Glucose** |
| --- | --- | --- |
| **Fraction of INS-1 cells responding to GW9508** | 58 of 74 (78%) | 53 of 62 (85%) |
| **Fraction of cells with a transient response** | 34 of 74 (46%) | 50 of 62 (81%)** |
| **Fraction of cells with a sustained response** | 40 of 74 (54%) | 21 of 62 (34%)* |
| **Lag time (sec.)** | 149.9 ± 13.0 | 115.6 ± 8.5* |
